# Individual differences in behavioral and cardiovascular reactivity to emotive stimuli and their relationship to cognitive flexibility in a primate model of trait anxiety

**DOI:** 10.3389/fnbeh.2014.00137

**Published:** 2014-04-24

**Authors:** Yoshiro Shiba, Andrea M. Santangelo, Katrin Braesicke, Carmen Agustín-Pavón, Gemma Cockcroft, Mark Haggard, Angela C. Roberts

**Affiliations:** ^1^Department of Physiology, Development and Neuroscience, University of CambridgeCambridge, UK; ^2^Behavioural and Clinical Neuroscience Institute, University of CambridgeCambridge, UK; ^3^Department of Psychology, University of CambridgeCambridge, UK

**Keywords:** trait anxiety, fear generalization, marmoset, cognitive flexibility, prefrontal cortex, biomarkers

## Abstract

High trait anxiety is a risk factor for the development of anxiety disorders. Like the disorders themselves high trait anxiety has marked phenotypic variation at the level of symptomatology and neural circuits, suggesting that there may be different symptoms and distinct neural circuits associated with risk for these disorders. To address these issues, it is essential to develop reliable animal models of trait anxiety in a non-human primate whose brain bears structural and functional similarity to humans. The present study investigated individual variation in responsivity to fearful and anxiety provoking stimuli in the common marmoset monkey. Seven out of 27 animals failed to display discriminative, conditioned cardiovascular and behavioral responses on an auditory fear discrimination task, similar to that seen in high anxious humans and rodents. Their heightened emotionality to a rubber snake was consistent with the hypothesis that they were high in trait-like anxiety. Evidence for phenotypic variation in the high anxiety group was provided by the finding that discrimination failure was predicted early in conditioning by either hyper-vigilant scanning to the cues or a reduction in blood pressure to the context, i.e., test apparatus. Given that high trait anxiety in humans can be associated with altered prefrontal cognitive functioning and previously we implicated the marmoset anterior orbitofrontal (antOFC) and ventrolateral prefrontal cortex (vlPFC) in negative emotion regulation, we also tested the marmosets on two tests of cognitive flexibility differentially dependent on these two regions. While the high anxious group did not differ overall in their perseverative performance, the two distinct phenotypes were differentially correlated with reduced perseverative responding on the OFC- and vlPFC-dependent flexibility tests. Together, this study provides a new model of trait anxiety in marmosets amenable to analysis of phenotypic variation and neural circuitry.

## Introduction

Fear and anxiety are adaptive responses, elicited by explicit and uncertain threat, respectively. However, in excess, as in humans with high trait anxiety, they are a significant risk factor for developing mood and anxiety disorders (Chambers et al., 2004; Sandi and Richter-Levin, 2009). Trait anxiety refers to a general tendency to perceive and react negatively in a wide variety of stressful situations (Gaudry et al., 1975). High trait-anxious individuals show enhanced attentional bias toward negative cues (Bradley and Mogg, 1999; Cisler and Koster, 2010), are more likely to interpret emotionally ambiguous stimuli as threatening (Mathews et al., 1989; Richards et al., 2002) and display impaired performance on prefrontal-dependent cognitive control tasks (Bishop, 2009; Visu-Petra et al., 2012). Consistent with these findings, a high anxiety phenotype has been associated with decreased prefrontal activity, increased amygdala activity (Indovina et al., 2011) and reduced functional (Bishop, 2007; Hahn et al., 2011) and structural connectivity between the two (Kim and Whalen, 2009), features common with a variety of anxiety disorders, including post-traumatic stress disorder (Shin et al., 2005; Killgore et al., 2013; Stevens et al., 2013), panic disorder (Thomas et al., 2001; Killgore et al., 2013) and specific phobia (Ahs et al., 2009; Killgore et al., 2013). Thus, the study of trait anxiety has the potential to provide important insights into the etiology and treatment of anxiety disorders (Sandi and Richter-Levin, 2009; Indovina et al., 2011).

Marked individual differences in responsivity to fearful- and anxiety-provoking stimuli have been reported in other animals, including rodents (Duvarci et al., 2009) and monkeys (Nelson et al., 2003). The development of these non-human models will be essential for establishing the causal relationship between the observed alterations in neural circuitry in high trait anxious individuals and their behavioral phenotype. In particular, it is important to develop models in monkeys in which prefrontal structure and connectivity patterns are similar to those in humans (Price, 1999; Burman et al., 2006; Roberts et al., 2007; Burman and Rosa, 2009; Yeterian et al., 2012). The common marmoset is a well established primate model for cognitive neuroscience and an emerging model for molecular studies since the completion of its genome and the first transgenic marmoset (Sasaki et al., 2009), making it an ideal species for studying the interaction between genes, environment and brain development in the context of behavioral risk factors for affective disorders. Thus, the present study investigated individual differences in the responsivity of this species to fearful and anxiety-provoking stimuli. Differences in fear conditioning, in particular, discriminatory fear conditioning, are associated with high trait anxiety in both humans (Grillon, 2002) and rats (Duvarci et al., 2009), and so marmosets received Pavlovian discriminatory fear conditioning, whereby one of two auditory cues was associated with aversive loud noise (Experiment 1). Simultaneous recording of cardiovascular activity and behavior provided a comprehensive measure of the emotional state. Their observed individual differences in cardiovascular and behavioral responsivity were then compared to their performance in another anxiety-provoking context, namely, exposure to a rubber snake (Barros et al., 2002; Izquierdo and Murray, 2004; Clara et al., 2008; Machado et al., 2009) (Experiment 2).

Finally, we also determined their cognitive performance on two distinct tests of prefrontal function. Whilst impairments in prefrontal function have been commonly reported in high trait anxious humans (Bishop, 2009; Visu-Petra et al., 2012), improvements have also been reported (Belsky et al., 2009; Homberg and Lesch, 2011), particularly in the absence of anxiety provoking stimuli. However, to our knowledge, there have been no reports of the cognitive abilities of high and low trait anxious rodents or monkeys. Thus, given our previous findings that implicated both the antOFC and vlPFC in the regulation of fear and anxiety (Agustín-Pavón et al., 2012), we investigated the animal's performance on two appetitive cognitive flexibility tests differentially dependent upon these two ventral PFC regions (Wallis et al., 2001; Man et al., 2009).

## Materials and methods

### General experimental design

**Figure 1:**
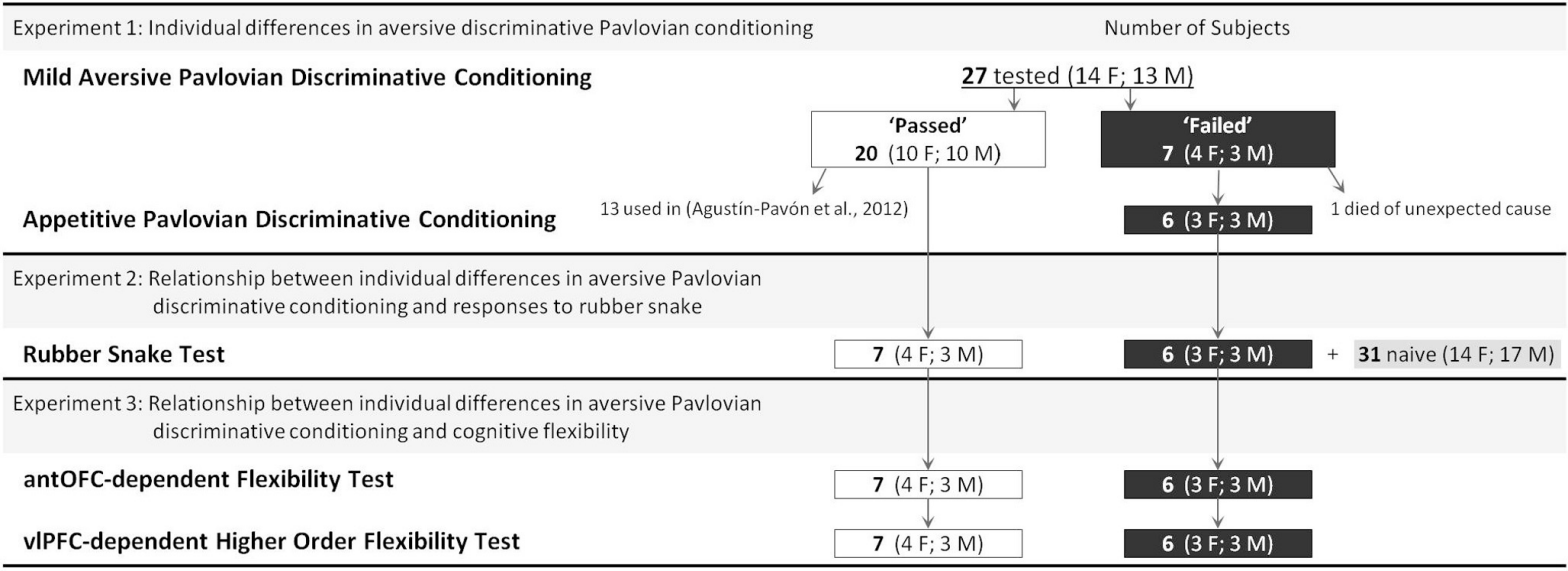
**General Experimental Schedule and Number of Subjects used for each Experiment**.

Figure 1 depicts the overall schedule and subject details. All procedures were approved by an Ethical Review Committee from the University of Cambridge and conducted in accordance with the project and personal licenses held by the authors under the UK Animals (Scientific Procedures) Act of 1986.

### Experiment 1: individual differences in aversive discriminative pavlovian conditioning

#### Subjects

Twenty-seven experimentally naïve common marmosets (*Callithrix jacchus*, 14 females and 13 males, average age 2.3 years ranging 1.6–3.1) were used (Figure 1). The animals were matured young adults in terms of both reproduction (Tardif and Smucny, 2003) and PFC morphology (Oga et al., 2013). The animals were housed in male/female pairs in rooms with controlled humidity and temperature and with a 12-h light/dark cycle. They were fed whole meal bread, hard-boiled egg, and a piece of fruit after testing. This diet was supplemented with additional fruit and nuts on the week ends. Water was available *ad libitum*.

#### Implantation of telemetry transmitter for cardiovascular recording

To measure heart rate (HR) and blood pressure (BP) changes remotely in animals, a PhysioTel Telemetry System (Data Sciences, St. Paul, Minnesota) was used. A telemetry transmitter (TA11PA-C40) was implanted into the abdominal cavity, and the probe catheter was inserted into the descending aorta as described previously (Braesicke et al., 2005).

#### Mild aversive pavlovian discriminative conditioning

***Test apparatus***. Behavioral testing took place within a sound-attenuated test apparatus. Each subject was transported from the home cage to the apparatus in a clear Perspex box. The carrying box, with the subject, was then fitted into the internal frame of the apparatus; a detailed illustration is given in (Mikheenko et al., 2010). Cardiovascular data were collected by the telemetric receiver (RPC-1, Data Sciences) placed underneath the floor of the internal frame. Sound conditioning stimuli were generated in AdobeAudition software (version 1.5) and played through a computer-controlled loudspeaker (Biotronix, UK). An unconditioned aversive noise stimulus was generated by a siren controller box (Electronics Development Group, Engineering Department, University of Cambridge) and played through a computer-controlled siren speaker (Biotronix, UK). The onset and offset of the light and sounds were controlled by Whisker device control software (Cardinal and Aitken, 2010).

***Test procedures***.

*Orienting*. Once the animals were habituated to the apparatus, showing relatively stable HR across three sessions, they were moved to the orienting sessions. Animals received two orienting sessions, in which two novel sounds, a 4 kHz tone and a clicker at 70 dB [the “to-be” conditioned stimuli (CSs)], were each presented pseudorandomly for a duration of 20 s, four times a session, on a variable inter-CS interval (icsi) schedule (120–180 s). The aim of these sessions was to monitor the behavioral and autonomic reactions of the animals toward the novel stimuli. The stimulus that elicited the smaller behavioral and autonomic reaction was chosen as the CS^+^, and the one that elicited the larger reaction became the CS^−^, thus avoiding any stimulus preparedness (Agustín-Pavón et al., 2012).

**Figure 2:**
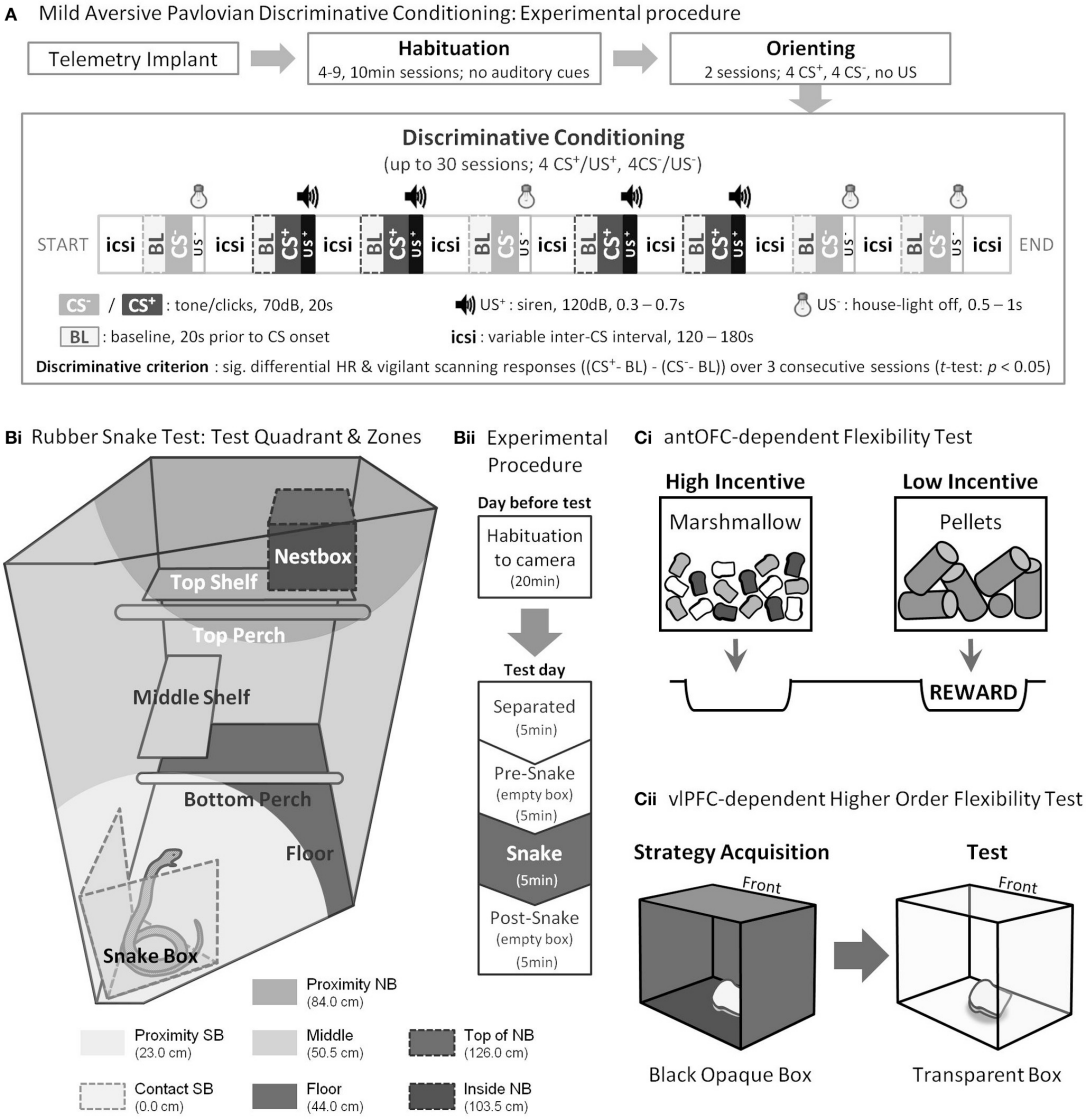
**Experimental Procedures**. **(A)** Pre-training and testing on the mild aversive Pavlovian discriminative conditioning paradigm. **(B)** Rubber snake test. **(Bi)** Top right-hand quadrant of the home cage viewed from the upper front corner and **(Bii)** Experimental procedure. **(C)** Cognitive flexibility tests. **(Ci)** In the antOFC-dependent flexibility test, to obtain the food reward, the subject was required to choose the box with low-incentive pellets whilst inhibiting their prepotent response for the high-incentive marshmallows. **(Cii)** In the vlPFC-dependent higher order flexibility test, the subject was required to transfer the acquired strategy from the opaque to the transparent box while inhibiting reaching directly to the now visible reward. “Front” denotes the surface of the box that was immediately facing the marmoset.

*Conditioning*. Following the orienting, the animals received Pavlovian conditioning in which one of two auditory cues (CS^+^) was associated with a burst of mildly aversive loud noise [unconditioned stimulus (US^+^), 120 dB, 0.3–0.7 s] and the other (CS^−^) with a non-aversive, very brief period of “light off” (US^−^, 0.5 s) as described fully in Figure 2A. The brief period of “light off” following the CS^−^ was used to increase the overall discriminative ability of the two CSs (Fedorchak and Bolles, 1986). In a session, 4 CS^+^s and 4 CS^−^s were pseudorandomly presented. The schedule and parameters were otherwise identical to those used in orienting. Each animal was given one session a day until they acquired the discriminative conditioning criterion (see below) or until they had received 30 sessions, whichever occurred first. The former was considered a successful, and the latter a failed discrimination.

*Discriminative criterion*. Each animal was given one session a day until vigilance behavior and heart rate during the CS^+^ (compared to baseline (BL)) was significantly greater than that to the CS^−^ (compared to BL) over three consecutive sessions (conditioning criterion) (Agustín-Pavón et al., 2012).

***Behavioral and cardiovascular measurement***. Behaviors during testing were video-recorded and subsequently scored by a research technician (GC) unaware of the experimental conditions and whether animals went on to pass or fail the discrimination. In addition, to assess the inter-rater reliability for the scoring, three sessions from each of two animals were randomly selected and scored by another experienced scorer. The intra-class correlation coefficient (ICC) was 0.75 [*F*_(35)_ = 6.93, *p* < 0.001] which is within the good range of reliability (Cyr and Francis, 1992). The CS^+^-related behaviors [typically displayed by marmosets in response to simple Pavlovian conditioning (Mikheenko et al., 2010)] were treated as a single measure of “vigilant scanning” and included attentive visual search of surroundings accompanied by tense postures marked by forward extension of body/head and rearing. The duration of the behavior displayed during the BL and CS periods was scored using a program written in QuickBASIC 4.5.

BP (systolic and diastolic) and HR data were recorded on a PC with data acquisition software Spike2 (version 7.01, Cambridge Electronic Design). Outliers (BP values >400 mmHg or <0 mmHg, or other abnormal spikes) were removed using an algorithm written in Visual Basic, and systolic and diastolic BP events were extracted as local minima and maxima for each heartbeat cycle as described previously (Agustín-Pavón et al., 2012). HR was the more reliable autonomic response to the CS^+^ both within and between animals (Mikheenko et al., 2010) and so together with behavior, was used for the discriminative criterion (Figure 2A).

#### Appetitive pavlovian discriminative conditioning

To rule out the possibility that any failure in fear discriminative conditioning was due to a general impairment in learning ability, six (3 female; 3 male) of the seven subjects that failed (one animal died of unexpected causes) were tested on an appetitive Pavlovian discriminative conditioning paradigm (Reekie et al., 2008). A similar experimental setting to that of the aversive conditioning paradigm was used, with the sound that was used as CS^+^ in the aversive conditioning paradigm staying as CS^+^ and the CS^−^ staying as CS^−^. However, instead of aversive loud noise, the CS^+^ was associated with reward (half-full box of marshmallows, US^+^) and the CS^−^ with the absence of reward (empty food-box, US^−^). Two-thirds of the sessions contained a CS^+^ along with 0–2 CS^−^s. The remainder of the sessions contained 1–2 CS^−^s only. The length of the CSs, icsi, and BL periods were the same as for the fear discrimination paradigm (see Figure 2A). The period of access to either the empty or half-full food-box was 2 min. Discriminative criterion was defined as significantly greater “head jerk” behavior (CS-directed orienting responses consisting of a flick/snap of the head) (Reekie et al., 2008) and BP to six consecutive CS^+^s compared to the intervening 6–14 CS^−^s.

#### Statistical analysis

All cardiovascular and behavioral data were analyzed with *t*-test, mixed design ANOVA and logistic regression analysis (SPSS versions 17–21). The behavioral data in the orienting session showed a violation of normality assumption (detected by Shapiro–Wilk test), therefore log transformation was performed prior to hypothesis testing.

### Experiment 2: relationship between individual differences in aversive pavlovian discriminative conditioning and emotional responses to a rubber snake

#### Rubber snake test

***Subjects***. Seven “passed” (4 female, 3 male) and six “failed” (3 female, 3 male) animals from Experiment 1 (see Figure 1). The remaining animals from Experiment 1 went on to receive lesions of the prefrontal cortex for another study (Agustín-Pavón et al., 2012).

***Stimulus***. A model snake made of rubber was used as a stimulus. It resembled a cobra and was coiled with its head raised (27 cm in height) and dark brownish in color with black stripes. A triangular prism box made of opaque white Perspex (26 × 26 × 29.5 cm triangle sides × 30 cm high) contained the rubber snake. By removing the sliding door, the snake could be revealed to the subject. The box was designed to conceal the snake from all marmosets except the target subject. The animals had never seen the snake or the box before the experiment.

***Test procedures***. Testing took place in the home cage following a habituation session the day before, which was identical to the test session except that the box did not contain the rubber snake. On the test session, the subject was first separated from the cage mate into the upper right quadrant (92 cm high × 60 cm wide × 98 cm deep, Figure 2Bi), preventing visual contact. The 20-min session was divided into four 5-min phases: “Separated” (only camera and microphone were present), “Pre-snake” (an empty box was placed in the test quadrant), “Snake” (the empty box was replaced with a box containing the rubber snake), and “Post-snake” (an empty box) (Figure 2Bii). Testing took place between 12:00 and 13:00 on week days. The order of testing was randomized across the animals.

***Behavioral measurements***. Video-recorded behaviors were scored by a person blind to the experimental conditions using a quantitative analysis program (JWatcher, Version 1.01). The vocalizations were observed only in the presence of the threat stimulus. They were recorded by a shotgun microphone (Pulse, NPM702) ensuring that the target animal's calls could easily be distinguished from any other animals' calls in the room. The calls were analyzed with sound spectrogram (Syrinx-PC software, Version 2.61). Behavioral parameters included:

1. Average distance from the snake. The test quadrant was divided into seven zones based on the proximity to the snake (Figure 2Bi). The proportion of time an animal spent in each zone over the 5-min phase was scored. The average distance was obtained by multiplying these proportions with the mean distance of each zone from the snake and summing the products.
2. Locomotion. The proportion of time an animal spent in translational movement over the 5-min phase. The translational movement was registered when an animal altered its body position using all four limbs.
3. Stare duration. The proportion of time an animal spent staring at the model snake. Staring was defined as any time when an animal's eyes and head were oriented directly toward the model snake.
4. Stare frequency. The number of discrete occasions an animal stared at the model snake.
5. Head-cock. Number of head movements from side to side while the animal's attention is directed to the model snake. This behavior has been reported as an observational behavior (Barros et al., 2002).
6. Tsik call. A short and loud “tsik” sound. It has been reported as an alarm/mobbing call (Cross and Rogers, 2006; Bezerra and Souto, 2008; Clara et al., 2008; Cagni et al., 2011).
7. Tsik-egg call. A tsik call closely followed by an egg call (a short call with a few harmonics). This call is associated with vigilance behavior (Pistorio et al., 2006; Bezerra and Souto, 2008).

***Statistical analysis***. All analyses were performed using SPSS (version 17–21). To provide a thorough characterization of the pattern of behavior displayed to the rubber snake and to maximize the sample size available for the subsequent principal component analysis (PCA), test data from additional 31 experimentally naïve marmosets (14 female, 17 male) were analyzed alongside the 13 “passed” and “failed” animals (see Figure 1). The latter fell within the observed range. For the “Snake” phase, PCA was performed (*n* = 44) to reduce the separate but correlated measures into weighted composites that reflect underlying psychological dimensions (Field, 2009). Component scores for individual animals were calculated using Anderson-Rubin method (Field, 2009) and used for subsequent mixed-design ANOVA and multiple regression (*n* = 13). Adequacy of sample size for PCA was assessed by the Kaiser-Meyer-Olin test, which returned an acceptable value of 0.64 (Field, 2009).

### Experiment 3: relationship between individual differences in aversive pavlovian discriminative conditioning and cognitive flexibility

#### Subjects

Seven “passed” (4 female, 3 male) and six “failed” (3 female, 3 male) animals (see Figure 1).

#### antOFC-dependent flexibility test

Animals were tested in a Wisconsin General Test Apparatus (WGTA) as previously described (Man et al., 2009). In each test trial (30 trials/session) they were presented with a choice between high-incentive marshmallows and low-incentive food pellets within transparent Perspex boxes. A response (touch) to either box resulted in the box being withdrawn, revealing the food well underneath. A response to the low-incentive, but not the high-incentive food box, was associated with food reward (syrup bread); thus, the subject was required to inhibit the prepotent response tendency to choose the high-incentive stimulus (Figure 2Ci). Testing was video-recorded and subsequently scored. Signal detection theory (Macmillan and Creelman, 2005) was used to classify the type of error responses into “perseverative” (responding to the incorrect stimulus significantly above chance) and “non-perseverative” (responding to the incorrect stimulus at or below chance) for each block of 10 trials (Clarke et al., 2004; Man et al., 2009).

#### vlPFC-dependent higher order flexibility test

Animals were tested in the WGTA as previously described (Wallis et al., 2001). Briefly, animals were first trained, on each trial, to touch and check each of three doors of a black opaque Perspex box in order to locate the unlocked door and retrieve the food reward (a piece of marshmallow) from within. Only one of the three doors of the box was unlocked on each trial, and success was defined as having found the unlocked door without having checked any door more than once. Having learned this strategy and performed 16 successful trials within a 21-trial session for four consecutive sessions, they progressed to the two test sessions. These were identical to training, except that a transparent box replaced the black opaque box. Although the subject could now “see” the reward along their direct line of sight (i.e., through the front door), the same strategy as before was required to obtain the reward (Figure 2Cii). A reach was defined as making contact with the door and then taking the hand away again. A failed reach (directly toward the now visible reward) to the locked front door was denoted a “barrier reach” error (Wallis et al., 2001), and was considered a sign of perseveration. Errors to the locked side door were denoted “non-barrier” reach errors.

#### Statistical analysis

Errors across groups were analyzed using *t*-test and factorial ANOVA. Correlation analysis and the Williams and Steiger test were performed to compare the measures from aversive discriminative conditioning with cognitive test performance.

## Results

### Individual differences in the ability to acquire mild aversive pavlovian discriminative conditioning

**Figure 3:**
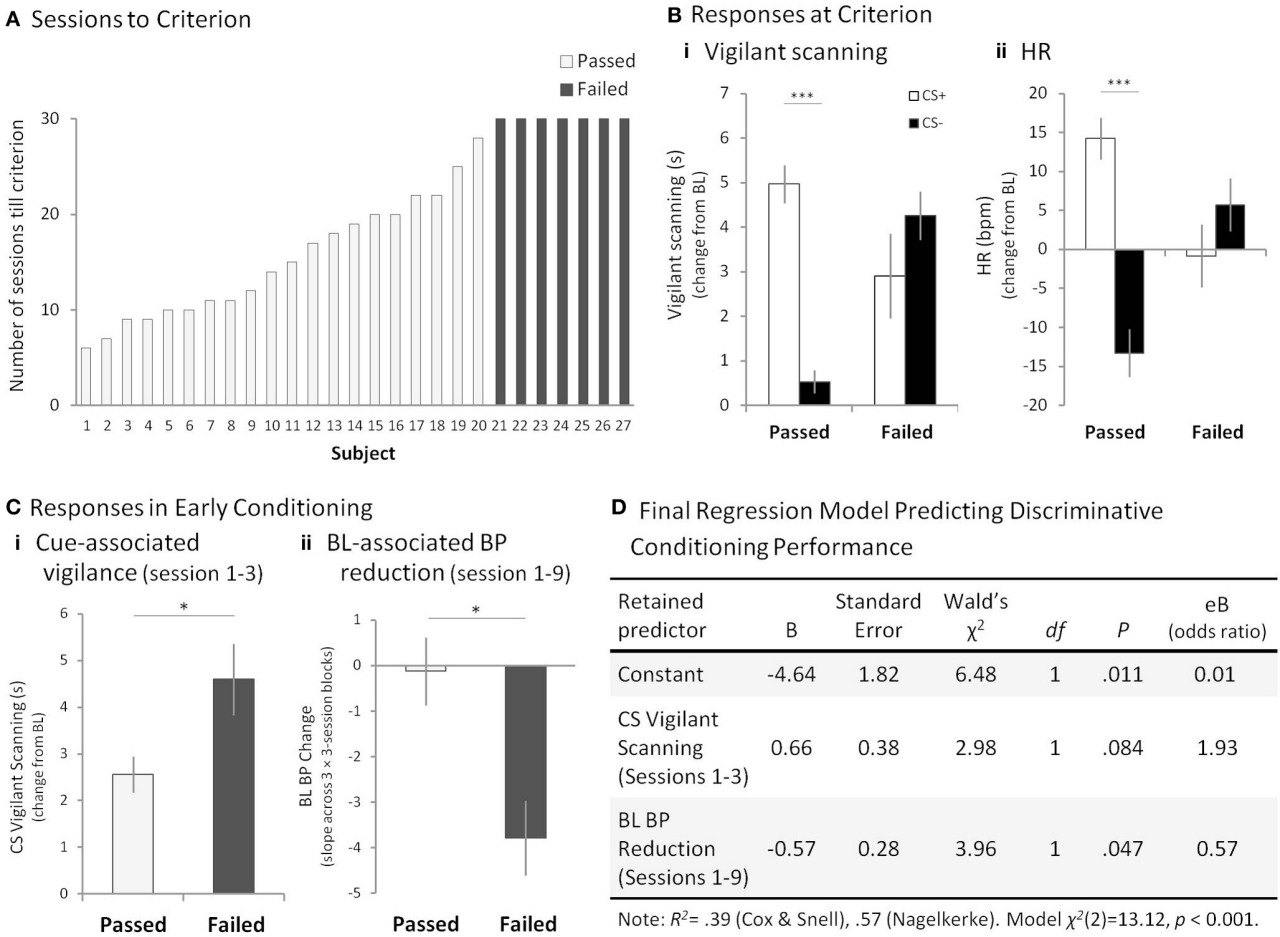
**Mild Aversive Pavlovian Discriminative Conditioning. (A)** The number of sessions that each subject in the “passed” (open bar) group (1–20) took to reach the criterion of significantly higher vigilant scanning and HR to the CS^+^ compared to the CS^−^, across a series of three consecutive sessions. The “failed” (filled bar) group (subjects 21–27) had still failed to reach criterion by the 30th session. **(B)** Responses to the CS^+^ and CS^−^, compared to BL in the three discrimination criterion sessions for the “passed” group and sessions 28–30 for the “failed group” for **(Bi)** mean vigilant scanning scores, and **(Bii)** mean HR. Error bars show standard error of the mean (SEM). **(C)** “Passed” and “failed” group comparison of **(Ci)** mean cue-associated vigilant scanning scores during sessions 1–3 and, **(Cii)** mean BL-associated hypotension measures during sessions 1–9. Error bars show SEM. **(D)** Of the three variables inserted into the logistic regression analysis, the cue-associated vigilance and BL-associated hypotension were retained in the final model as predictors of passing or failing the aversive discrimination. The positive coefficients for the CS vigilant behavior in sessions 1–3 indicated that as the vigilant behavior score increased by one unit, the odds of failing the discrimination increased from 1.0 to 1.93. On the other hand, the negative coefficient β indicated that as the BL BP measure declined by one unit, the odds of passing the discrimination decreased from 1.0 to 0.57. Thus, the greater BL BP decline, the more likely the animal was to fail the discrimination. ^*^p < 0.05 for “passed” vs. “failed,” ^***^p < 0.001 for CS^+^ vs. CS^−^.

After repeated exposure to the CS^+^ associated with the aversive loud noise and to the CS^−^ associated with the neutral stimulus, 20 out of 27 animals (74%) developed significant discriminative conditioned behavioral [CS^+^ vs. CS^−^, *t*_(22)_ > 2.14, *p* < 0.05, for each animal] (Supplementary Table 1, “Passed”) and cardiovascular responses [*t*_(22)_ > 2.12, *p* < 0.05, for each animal] (Supplementary Table 2, “Passed”), between the 6th and 28th session, thus passing the criterion. The remaining seven animals (26%), however, failed to attain the discriminative conditioned responses even after 30 sessions (exposure to 120 CS^+^s and CS^−^s each) [behavior: *t*_(22)_ < 1.21, HR: *t*_(22)_ < 1.49, for each animal] (Figure 3A, Supplementary Tables 1, 2, “Failed”). We compared the mean vigilant scanning toward the CSs in the three criterion sessions of the “passed” group with the final three sessions (i.e., 28–30) of the “failed” group. This showed that the “passed” group displayed significantly greater vigilance to the CS^+^ compared to the CS^−^, whereas vigilance was heightened but did not differ between the CSs in the “failed” group [Two-Way factorial ANOVA: Group × CS, *F*_(1, 25)_ = 47.29, *p* < 0.001; *post-hoc* pairwise comparison of CS^+^ vs. CS^−^ for “passed,” *F*_(1, 25)_ = 106.95, *p* < 0.001, for “failed,” *F*_(1, 25)_ = 3.50, *p* = 0.073] (Figure 3Bi). Similarly, the “passed” but not the “failed” group also showed heightened HR during the CS^+^ compared to the CS^−^ [Group × CS, *F*_(1, 25)_ = 46.04, *p* < 0.001; *post-hoc* pairwise comparison of CS^+^ vs. CS^−^, for “passed,” *F*_(1, 25)_ = 115.72, *p* < 0.001, for “failed,” *F*_(1, 25)_ = 2.31, *p* = 0.141] (Figure 3Bii).

### Cardiovascular and behavioral responses early in training together predict eventual success or failure in aversive discriminative conditioning

Next, we determined whether individual differences in behavioral and cardiovascular reactivity in the early sessions were associated with eventual success or failure to display discriminative conditioning. This comparison was made at a time-point well before the majority of animals had shown any evidence of cardiovascular or behavioral conditioning and enabled us to see whether there were any early behavioral or cardiovascular biomarkers that would predict eventual discriminative failure. Responses during cue presentation (CSs) and BL were averaged across the first three sessions and compared between the “passed” and “failed” groups. This revealed that animals in the “failed” group displayed significantly greater vigilance responses to both CSs [Two-Way factorial ANOVA (Group × CS): Group, *F*_(1, 25)_ = 6.71, *p* < 0.05] (Figure 3Ci, Supplementary Figure 1A) and significantly greater HR responses to the CS^−^ compared to those that passed (“passed”: Mean (*M*) = 2.37, Standard Error of Mean (SEM) = 1.72, “failed”: *M* = 14.14, SEM = 5.34) [Group × CS: *F*_(1, 25)_ = 11.57, *p* < 0.01; *post-hoc* pairwise comparison: “passed” vs. “failed,” for CS^+^, *F*_(1, 25)_ = 0.25, *p* = 0.619, for CS^−^, *F*_(1, 25)_ = 7.75, *p* < 0.01] (Supplementary Figure 1B). Such increased vigilance and HR to the CSs were not seen in the pre-conditioning orienting sessions in the absence of aversive stimulus although there was a trend for increased vigilant scanning to the CSs [behavior: *F*_(1, 25)_ = 3.26, *p* = 0.08, HR: *F*_(1, 25)_ = 0.62, *p* = 0.44]. Whilst there were no differences between the groups in BL cardiovascular responses in the first three sessions, there was a noticeable decline in BL HR (bradycardia) and BP across the next few sessions, reaching a nadir by session 9, in the animals that failed the discrimination (Supplementary Figures 1C,D). The slope of the best fitting line over the mean of three, three-session blocks (1–3, 4–6, 7–9) was used to quantify this decline; the negative value indicating reduction in BP [One-Way ANOVA of the slopes across sessions: “passed” vs. “failed,” for BP, *F*_(1, 25)_ = 7.27, *p* < 0.05, for HR, *F*_(1, 25)_ = 4.24, *p* = 0.05] (Figure 3Cii). No such baseline differences were observed in vigilant scanning [*F*_(1, 25)_ = 1.60, *p* = 0.218].

To assess how reliably the responses in the early sessions predicted the eventual success or failure of the animals in discriminative conditioning, a binary logistic regression analysis was performed using, as predictor variables, all behavioral and cardiovascular measures that showed highly significant group differences in the early sessions (CS vigilant scanning, HR CS^−^, BL hypotension). The final model [χ^2^_(2)_ = 13.12, *p* < 0.001; backward stepwise strategy] retained two of the variables, namely, CS vigilant scanning and BL hypotension as significant predictors (Figure 3D).

### Animals that fail to acquire aversive discriminative conditioning do acquire appetitive discriminative conditioning

Despite failing to discriminate the CSs in the aversive discrimination paradigm, all of the “failed” group animals (*n* = 6) successfully acquired discriminatory behavioral (head jerks) and cardiovascular (BP) responses to the same CSs in the appetitive condition [CS^+^ vs. CS^−^, behavior: *t*_(9–18)_ > 2.26, *p* < 0.05, BP: *t*_(9–18)_ > 2.17, *p* < 0.05, for each animal] (Supplementary Figures 2A,B), with a mean number of 14.33 (SEM = 2.85) CS^+^ trials to reach the criterion. Moreover, their rate of learning was within the normal range of all other marmosets in the colony previously trained on this appetitive task (*n* = 23, *M* = 16.35, SEM = 2.12) (Supplementary Figure 3).

### Animals that fail to acquire aversive discriminative conditioning exhibit a heightened anxiety-related emotional response to a rubber snake

**Figure 4:**
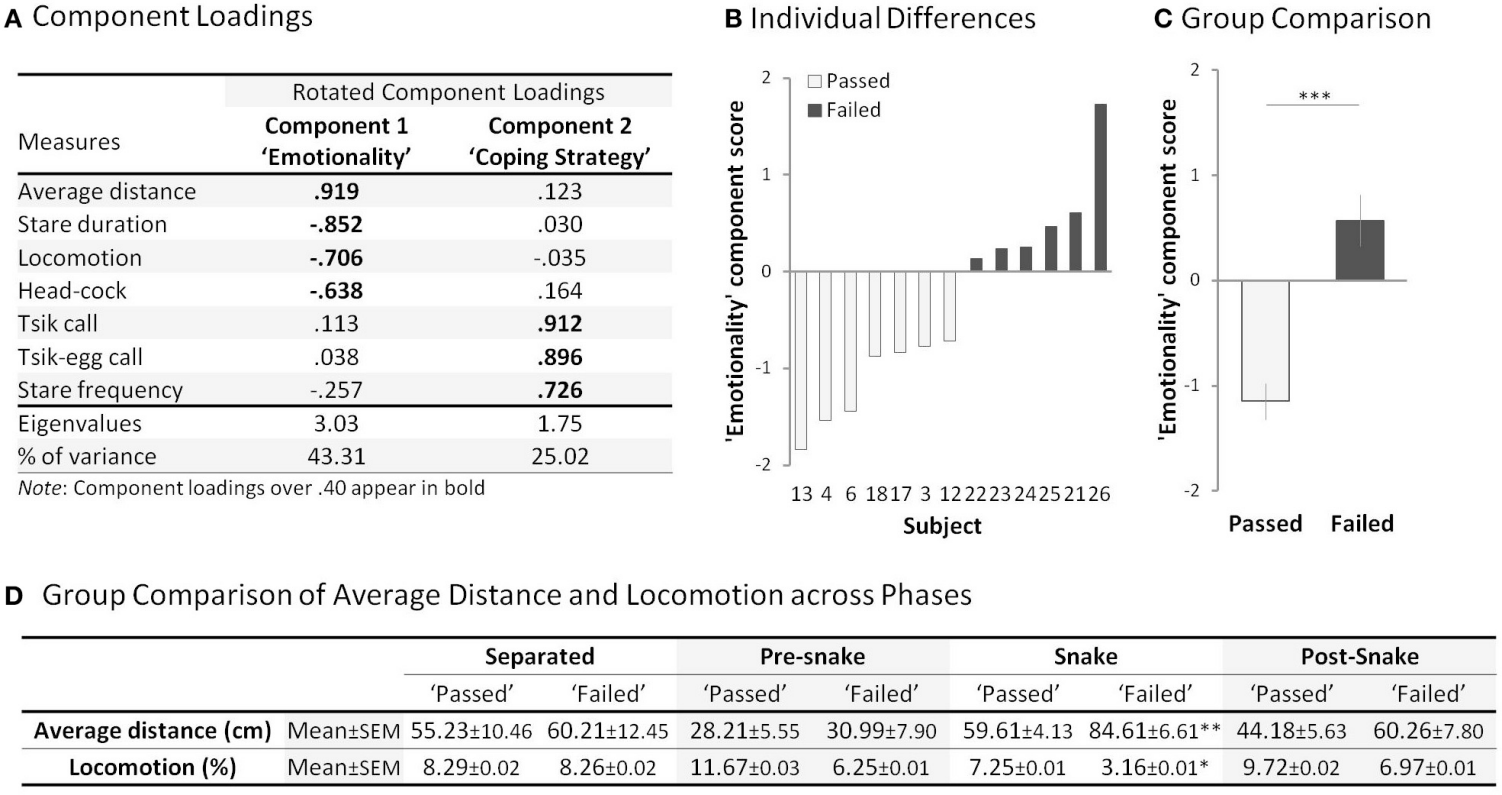
**Rubber Snake Test. (A)** Component loadings of the measures in the rubber snake test (*n* = 44). **(B)** “Emotionality” (principle component 1) score of each subject in the “passed” (open bar) and “failed” (filled bar) groups. Subject numbers correspond to the numbers in Figure 3A. **(C)** Comparison of mean “Emotionality” component scores between the “passed” and “failed” groups. Error bars show s.e.m. **(D)** Mean average distance and locomotion measures of the “passed” and “failed” groups across the four phases. ^*^p < 0.05, ^**^p < 0.01, ^***^p < 0.001 for “passed” vs. “failed.”

When presented with a rubber snake, the “failed” group displayed behavioral patterns that indicated heightened fear and anxiety, compared to that of the “passed” group (Supplementary Table 3). A PCA revealed two principal components (rotated; with an eigenvalue over 1.00) accounting for 68.33% of the total variance (Figure 4A). The variables loading highly on component 1 included distance from the snake, total stare duration, locomotion and number of head-cocks. Animals with higher component 1 scores maintained a considerable distance from the snake, avoided staring at the snake, displayed reduced locomotion and head-cocks. These are indicative of high anxiety/emotionality, a pattern of behavior displayed by the “failed” group [*F*_(1, 11)_ = 35.24, *p* < 0.001] (Figures 4B,C). The behaviors loading highly on component 2 were stare frequency and tsik and tsik-egg calls that are emitted only in the presence of a predator threat and used to drive the threat away (Bezerra and Souto, 2008; Clara et al., 2008). Animals with higher component 2 scores emitted greater numbers of calls and displayed a higher frequency of short latency “looks” at the snake, behaviors which are hypothesized overall to contribute to a proactive coping strategy (Koolhaas et al., 1999; Cross and Rogers, 2006). This measure did not differ significantly between the groups (“passed”: *M* = 0.37, SEM = 0.30, “failed” *M* = 1.18, SEM = 0.64) [*F*_(1, 11)_ = 1.45, *p* = 0.254] (Supplementary Figure 4). Nor did the groups differ in their distance and locomotion scores in the other test phases in the absence of the snake [Average distance: “Separated” *t*_(11)_ = −0.31, *p* = 0.763; “Pre-snake” *t*_(11)_ = −0.29, *p* = 0.774; “Snake” *t*_(11)_ = −3.31, *p* < 0.001; “Post-snake” *t*_(11)_ = −1.71, *p* = 0.116, Locomotion: “Separated” *t*_(11)_ = 0.01, *p* = 0.993; “Pre-snake” *t*_(11)_ = 1.77, *p* = 0.105; “Snake” *t*_(11)_ = 2.38, *p* < 0.05; “Post-snake” *t*_(11)_ = 1.06, *p* = 0.311] (Figure 4D).

### Cue-associated hyper-vigilance and BL-associated reduction in BP are differentially correlated with reduced perseveration on antOFC- and vlPFC-dependent cognitive flexibility tests

**Figure 5:**
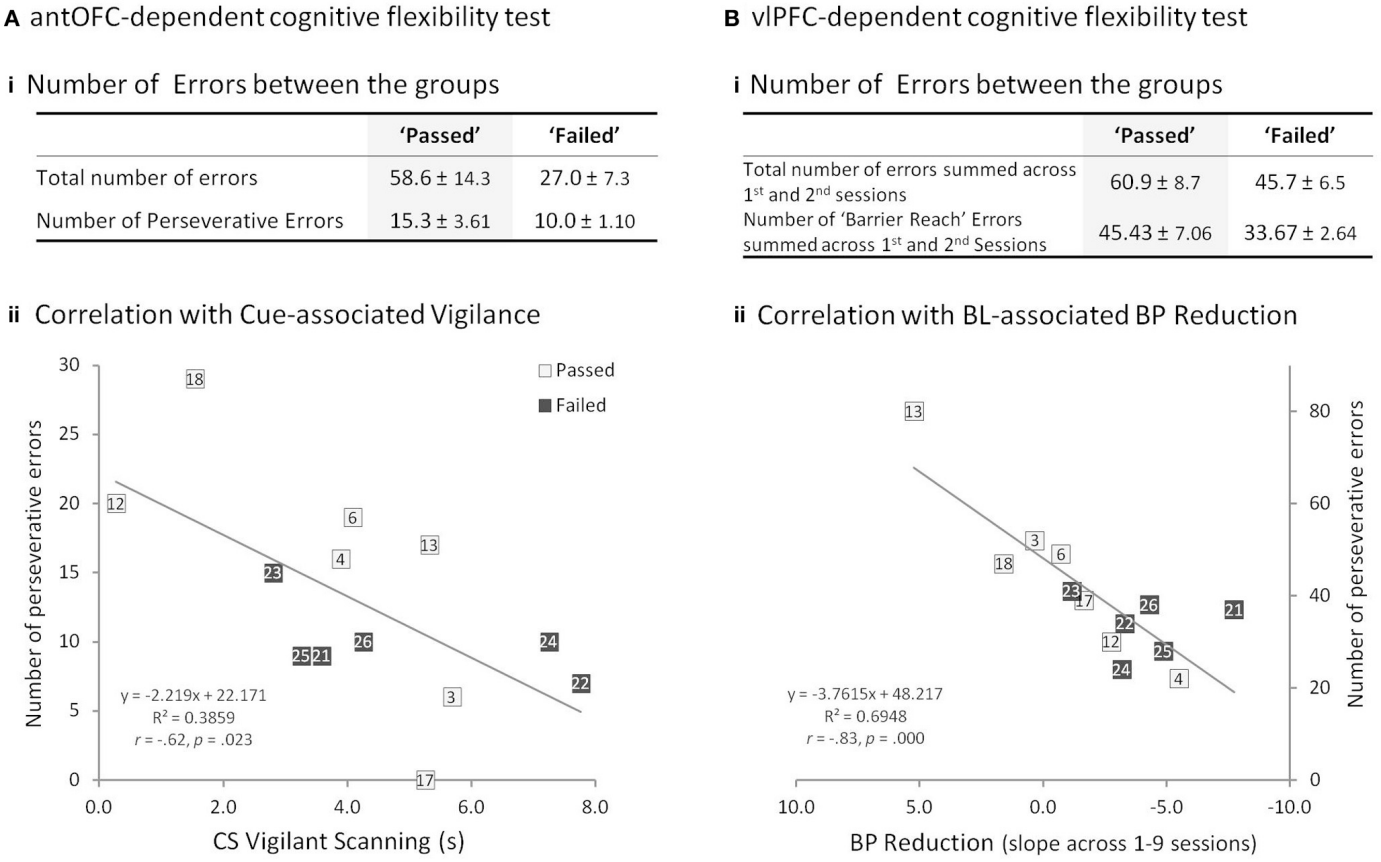
**Prefrontal-dependent Flexibility Tests. (A)** antOFC-dependent cognitive flexibility test. **(Ai)** Comparison of “passed” and “failed” groups for the total number of errors and the number of perseverative errors (mean ± s.e.m.). No significant group difference. **(Aii)** Significant negative correlation between the number of perseverative errors in the antOFC-dependent test and cue-associated vigilant scanning scores of the animals in the “passed” (open square) and “failed” (filled square) groups. Numbers in the squares correspond to the subject numbers in Figure 3A. **(B)** vlPFC-dependent cognitive flexibility test. **(Bi)** Comparison of “passed” and “failed” groups for the total number of errors (both “barrier reach” and “non-barrier reach” errors) and the number of “barrier reach” errors summed across the first and second sessions. **(Bii)** Significant negative correlation between the number of perseverative “barrier reach” errors in the vlPFC-dependent test and BL-associated BP reduction of the animals in the “passed” and “failed” groups. The x-axis is flipped so that those with greater BP reduction are seen on the right side of the graph.

In the antOFC-dependent flexibility test, all animals successfully learned to inhibit a prepotent response tendency and select the low-incentive food-box, rather than the high-incentive food-box. Comparison of the “passed” and “failed” groups for the total number of errors to reach the discrimination criterion returned a trend level difference [*t*_(11)_ = 1.87, *p* = 0.089], this was due to a tendency for the “failed” group to make fewer errors (Figure 5Ai). There was considerable individual variation in the number of perseverative errors, a measure that has been shown to be affected by OFC lesions (Man et al., 2009), but this measure did not significantly differ between “passed” and “failed” groups [*t*_(11)_ = 1.31, *p* = 0.219] (Figure 5Ai).

Likewise, in the vlPFC-dependent flexibility test, all animals learned to refrain from reaching directly toward the visible food reward and instead, extrapolate the detour reaching rule from the opaque to the transparent box. Despite marked individual variation, “passed” and “failed” groups did not significantly differ in either the total number of errors (number of both “barrier reach” and “non-barrier reach” errors across first and second sessions) or the perseverative errors (number of “barrier reach” errors across first and second sessions), a measure that has been shown to be affected by vlPFC lesions (Wallis et al., 2001) [Three-Way factorial ANOVA of group × error type (“barrier reach”/“non-barrier reach”) × session (1st/2nd): no main effect of group, *F*_(1, 11)_ = 2.31, *p* = 0.157; no group × error type interaction, *F*_(1, 11)_ = 1.29, *p* = 0.281; no group × error type × session interaction, *F*_(1, 11)_ = 0.00, *p* = 0.954] (Figure 5Bi).

The perseverative measures from the antOFC-dependent and vlPFC-dependent tasks did not correlate with each other [Person's *r* = 0.21, *p* = 0.50] (Supplementary Figure 5), which is consistent with the finding that performance on these two tests is differentially dependent upon two distinct regions of vPFC (Wallis et al., 2001; Man et al., 2009). However, perseverative responding on the two tests did correlate, differentially, with the two variables that predicted failure on the discrimination task. Specifically, perseverative responding on the antOFC-dependent test correlated negatively with cue-associated vigilance [Pearson's *r* = −0.62, *p* < 0.05] (Figure 5Aii), but not with BL-associated reduction in BP [*r* = 0.37, *p* = 0.21] (Supplementary Figure 6) and importantly, the two correlations were significantly different from one another [Williams and Steiger test; *t* = 3.38, *p* < 0.01]. Conversely, perseverative responding on the vlPFC-dependent test correlated negatively with BL-associated reduction in BP [Pearson's *r* = −0.83, *p* < 0.001] (Figure 5Bii), but not with cue-associated vigilance [Pearson's *r* = 0.08, *p* = 0.80] (Supplementary Figure 7), and again the Williams and Steiger test confirmed that the correlations were significantly different [*t* = 2.84, *p* < 0.05]. Thus, the animals that displayed a heightened vigilance response to the CSs made fewer perseverative errors specifically in the antOFC-dependent test, and those that displayed BL-associated reduction in BP made fewer perseverative responses specifically in the vlPFC-dependent test.

## Discussion

In a cohort of experimentally naive marmosets tested on a mild aversive discriminative conditioning paradigm, seven out of the 27 animals failed to show discriminative conditioned responses between the danger cue, CS^+^, and safety cue, CS^−^(Experiment 1). Consideration of their behavioral and cardiovascular responsivity during the early stage of discrimination training (before learning in the majority of animals had taken place) revealed that failure to discriminate was predicted either by a display of hyper-vigilance to both cues (CS^+^ and CS^−^) and/or the development of reduced BP during the BL. Poor general learning abilities offer an unlikely explanation for the observed failure, given the animals in the “failed” group showed intact ability to acquire such discriminative responses in an appetitive setting. Instead, their failure is more likely due to high trait anxiety given that the “failed” group also showed heightened emotionality during the presentation of a predatory stimulus, a rubber snake, compared to the “passed” group (Experiment 2). Subsequent testing on two cognitive flexibility tests revealed an association between the two distinct behavioral and cardiovascular predictors of failed performance on the discrimination test and flexible cognitive performance (Experiment 3). Reduced perseverative performance was highly correlated with cue-associated hyper-vigilance on the antOFC-dependent flexibility task and with the BL-associated reduction in BP on the vlPFC-dependent flexibility task.

### Anxiety-induced fear generalization

Fear generalization is a feature of high trait anxiety (Reiss, 1997; Grillon, 2002) and a key symptom of clinical anxiety (Dunsmoor et al., 2011). In particular, a failure to display discriminative conditioned responses, and over-generalize instead, has been reported in patients suffering from panic disorder (Lissek et al., 2010), post-traumatic stress disorder (Grillon and Morgan, 1999; Mauchnik et al., 2010), and generalized anxiety disorder (Lissek et al., 2013). In comparison to relatively well documented studies in humans, very few studies involving animal models have addressed the association between failure to display discriminative conditioned responses and trait anxiety, apart from Duvarci et al. (2009) which reported a discriminative conditioning failure among rats with a high anxiety phenotype, as measured by performance on the elevated plus maze.

In the present study, even after a lengthy period of training, seven animals (26%) were unable to discriminate between the CS^+^ and CS^−^. It is unlikely that this reflects poor learning ability in general, as the same seven animals learned to discriminate between the same CS^+^ and CS^−^ when presented in an appetitive setting. Instead, their failure was more likely the result of fear generalization, either to the CS^−^ (or safety signal) or to the overall context of the apparatus. Evidence for generalization of conditioned responses to the CS^−^ is 2-fold. First, by the end of conditioning the “failed” group displayed a similar magnitude of vigilance responses to both CSs. Second, upon initial exposure to the aversive loud noise (first three sessions) the animals that eventually failed to discriminate, displayed increased vigilant scanning and HR responses to both the CS^+^ and CS^−^ compared to the “passed” group, indicative of heightened emotionality in the presence of the aversive loud noise. In contrast, heightened responses to the same stimuli were not seen in the prior orienting sessions in which there was no aversive loud noise, although there was a trend for the “failed” group to show heightened vigilant scanning even in this period. These findings are consistent with the observation that high anxious individuals can show impairments in inhibiting fear responses to a safety signal (Grillon and Ameli, 2001). However, animals that subsequently failed the discrimination were also more likely to show a selective slowing of HR and decrease in BP during the BL period, across the first few conditioning sessions, compared to those that passed. Responses in the BL of a conditioning task usually reflect associative learning about the context, i.e., the apparatus in which the animal receives the unconditioned aversive stimulus, as distinct from any specific cues (Morgan and LeDoux, 1999; Grillon, 2008). Thus, the slowing of HR and decrease in BP that developed in the BL over the first nine sessions, as the “failed” animals received more and more pairings of the aversive loud noise, likely reflects generalized responding to the conditioning context. Whilst increased anxiety may have been predicted to induce increases rather than decreases in BP, a recent series of studies have reported associations between anxiety and lowered BP in young and elderly humans (Hildrum et al., 2008, 2011) and similarly, a negative association between worry-prone individuals and BP (Delgado et al., 2013).

Regression analysis revealed that of these different behavioral and cardiovascular measures relating to generalized responding to the safety cue and the context, heightened vigilant scanning to the CS^−^ and reduced BP in the BL were the best predictors of discrimination failure, and were better together than on their own. Thus, these measures acted as biomarkers for eventual discrimination failure. In addition, these findings suggest that some animals may have failed to discriminate because they generalized their fear responses to the CS^−^ whilst others may have failed to discriminate because they generalized their fear responses to the context. The hypothesis that such fear generalization was a consequence of being high in trait anxiety, was supported by the finding that this same group of animals showed increased emotionality in a completely distinct fear-provoking context, namely exposure to a rubber snake. Fear of snakes has been widely exploited in tests of anxiety both in humans and non-human primates (Öhman and Mineka, 2001). In marmosets, snakes are known to be their principle predators (Correa and Coutinho, 1997), and both captive-born and wild animals are known to exhibit a wide variety of emotive and defensive behaviors in their presence (Barros et al., 2002; Cross and Rogers, 2006; Clara et al., 2008; Cagni et al., 2011). In the present study the snake was presented in the animal's home cage environment surrounded by other conspecifics maximizing the ethological validity of the test. It should be noted that under these circumstances it cannot be ruled out that the animal's behavior to the snake may also have been influenced by the response of conspecifics. However, only the target animal could “see” the snake and thus any response of conspecifics was only in relation to the target animal's own behavior.

Upon encountering the snake stimulus, the animals that failed the discrimination, maintained a greater distance from the snake, showed reduced locomotion, as well as reduced numbers of head-cocks and stare duration in comparison to the animals that passed the discrimination. In contrast, large numbers of head-cocks and prolonged stare duration were apparent in those animals that approached the snake and appear indicative of an investigative response. The finding that these measures were diminished in those animals that maintained the greatest distance from the snake likely reflects their overall heightened avoidance of the aversive stimulus. When all behavioral measures were placed into a principle component analysis, the variables that were apparently indicative of avoidance and anxiety were reduced into one component, which was accordingly labeled “emotionality.” The “emotionality” scores were significantly higher in the “failed” animals than the “passed” animals. Thus, we demonstrate that the same animals displayed heightened emotionality across two very different paradigms, one involving learned fear and the other innate fear, consistent with the characteristics of a high anxiety trait. Moreover, the finding that some animals high in trait anxiety generalized to the safety cue in the fear discrimination task whilst others generalized to the context, suggests the existence of phenotypic variation within the high anxiety group, consistent with recent findings in rhesus monkeys with a high anxious temperament (Shackman et al., 2013).

### Relationship of trait-like anxiety with PFC-dependent cognitive flexibility

Having established a model of trait-like anxiety in marmosets, the cognitive abilities associated with this trait were investigated. Previous studies of trait anxiety in humans have implicated altered functioning within the PFC. In particular, deficits in attentional mechanisms (Bishop et al., 2004), working memory and inhibitory control (Eysenck and Calvo, 1992; Fox, 1994) associated with dorsolateral PFC have been identified in individuals high in trait anxiety, primarily in the presence of anxiety provoking stimuli (but see Bishop, 2009). However, contrary findings have been reported in humans with the short allele of the serotonin transporter polymorphism, a gene associated with anxiety-related traits (Lesch et al., 1996; Hariri et al., 2005) and enhanced vulnerability to developing mood and anxiety disorders as a consequence of early-life stress (Caspi et al., 2003). In this case, improvements on certain prefrontal dependent tests have been reported. This has led to the proposal that the short allele is associated overall with an enhanced sensitivity to motivationally relevant stimuli (Belsky et al., 2009; Homberg and Lesch, 2011), which, in the case of positive stimuli can lead to improvements in performance, in contrast to the impairments seen for negative stimuli. Improvements, rather than impairments in response inhibition have also been reported during sustained anxiety (Robinson et al., 2013) in humans. In the present study we chose to investigate cognitive functioning dependent upon appetitive stimuli related to the ventral regions of PFC because (i) we have implicated ventral regions, in particular the OFC and vlPFC in marmosets, in the regulation of conditioned fear and anxiety (Agustín-Pavón et al., 2012) and (ii) altered functioning in vlPFC has been reported in humans with high trait anxiety performing a fear discrimination task (Indovina et al., 2011).

There were no overall significant differences in performance on the OFC- and vlPFC- dependent flexibility tests between those animals that displayed discriminative fear conditioning (“passed” group) and those that had not (“failed” group); despite there being a tendency for the latter, high anxious group to make fewer perseverative responses on both tests. The finding that perseverative responding on the two tests did not correlate with one another is not surprising since we have shown previously that the performance of marmosets on these two tests is dependent on two distinct ventral regions of PFC. However, detailed inspection of the data suggested that those animals that had shown the greatest generalization to the safety cue on the fear discrimination task (i.e., heightened cue-associated vigilance) displayed fewer perseverative errors on the appetitive OFC-dependent task. Of note, hyper-vigilance has also been proposed to increase sensitivity to both negative *and* positive events in the *s*-allele carriers of the anxiety-related serotonin transporter gene (Belsky et al., 2009; Homberg and Lesch, 2011). In contrast, those animals that showed generalization to the context (i.e., context-associated reduction in BP) displayed fewer perseverative errors on the appetitive vlPFC-dependent test. Importantly, these effects were doubly dissociable, i.e., hyper-vigilance was correlated with OFC-dependent task performance and not with vlPFC-dependent task performance, and BP reduction was correlated with vlPFC-dependent task performance and not with OFC-dependent task performance. These findings support the hypothesis that the high anxiety trait may be associated with two distinct phenotypes and raises the intriguing possibility that these phenotypes may be related to altered functioning in distinct cognitive circuits associated with cued vs. contextual conditioning. Clearly such a hypothesis needs further testing with much larger n's than in the present study and with paradigms focusing on fear-related cues and contexts. However, these findings do resonate, not only with evidence of phenotypic variation such as cortisol level and socio-emotional behaviors associated with altered neural activity in rhesus monkeys with high anxious temperament (Shackman et al., 2013) but also in a recent study proposing two independent neurocognitive dimensions underlying trait anxiety in humans (Indovina et al., 2011). Finally, these results also provide preliminary evidence that under some circumstances high trait-like anxiety may be associated with improved cognitive performance (as reported in humans) since those animals that displayed the greatest tendency to show generalized responding on the discrimination test (which were also those animals that showed greatest emotionality in response to the snake) had a tendency to show reduced perseverative responding, i.e., improved flexibility.

Studies of trait anxiety have so far revealed altered interactions not only between the ventral PFC and the amygdala (Indovina et al., 2011) and bed nucleus of the stria terminalis (Fox et al., 2010) but also the medial PFC and the amygdala (Kim et al., 2011). Changes in hippocampal metabolic activity have also been associated with an anxious temperament (Oler et al., 2010). Evidence is also accruing for a role of many of these same circuits in discriminative fear conditioning, especially in terms of safety signal learning (Kazama et al., 2012; Kong et al., 2014). Although other brain regions have also been implicated in safety learning including the insula (Christianson et al., 2011; Kong et al., 2014) and striatum (Schiller et al., 2008), whether changes in these structures are also associated with trait anxiety is less clear. Future studies should determine the differential contribution of these distinct prefronto-limbic-striatal circuits to emotion regulation especially in terms of their selective contributions to affective processing vs. more general contributions to information processing and decision making *per se*. Along with the recognition that altered activity within these distinct circuits underlie phenotypic variation within the high anxiety personality trait, such studies may provide insight into the nosology of anxiety and mood disorders.

In summary, we have identified individual differences in trait-like anxiety in marmosets based on their conditioned behavioral and cardiovascular fear responses on a Pavlovian discrimination task and their innate emotional responsivity to a rubber snake. Those marmosets that failed to discriminate between a conditioned fear and safety cue, and generalized instead to either the safety cue or the overall context, also displayed significantly greater anxiety and avoidance responses to the rubber snake. Regression analysis suggested the existence of two possible phenotypes within the high anxious group. Comparison of performance on two tests of cognitive flexibility dependent upon the OFC and vlPFC, respectively, revealed no significant differences. However, perseverative responding on the two tests correlated differentially with the cue-associated hyper-vigilance and context-associated reductions in BP displayed by the high anxious group on the discrimination task, further supporting the existence of phenotypic variation. We propose that this model will facilitate the study of distinct symptomatology and neural circuitry underlying trait anxiety, of relevance to our understanding of the nosology of anxiety disorders.

### Conflict of interest statement

The authors declare that the research was conducted in the absence of any commercial or financial relationships that could be construed as a potential conflict of interest.
