# Supplementary material for: Individual differences in behavioral and cardiovascular reactivity to emotive stimuli and their relationship to cognitive flexibility in a primate model of trait anxiety
Source: Front Behav Neurosci. 2014 Apr 24;8:137. doi: 10.3389/fnbeh.2014.00137 (PMC4006051; doi:10.3389/fnbeh.2014.00137)
Supplement: Supplementary file 1 [file DataSheet1.DOCX]

***Supplementary Material***

**Individual differences in behavioral and cardiovascular reactivity to emotive stimuli and their relationship to cognitive flexibility in a primate model of trait anxiety.**

**Yoshiro Shiba^1,2^*, Andrea M. Santangelo^1,2^, Katrin Braesicke^1,2^, Carmen Agustín-Pavón^1,2^**, Gemma Cockcroft^2,3^, Mark Haggard^3^, Angela C. Roberts^1,2^**

^1^Department of Physiology, Development and Neuroscience, University of Cambridge, Downing Street, Cambridge, CB2 3DY, UK

^2^Behavioural and Clinical Neuroscience Institute, University of Cambridge, Downing Street, Cambridge, CB2 3EB, UK

^3^Department of Psychology, University of Cambridge, Downing Street, Cambridge, CB2 3EB, UK

* **Correspondence**: Yoshiro Shiba, Department of Physiology, Development and Neuroscience, University of Cambridge, Downing Street, Cambridge, CB2 3DY, UK

ys341@cam.ac.uk

** Current address: Department of Life Sciences, Imperial College London, South Kensington Campus, London SW7 2AZ, UK

1. **Supplementary Figures and Tables**

## Supplementary Tables

**Supplementary Table 1.** Individual animal’s mean vigilant behavior score for CS^+^, CS^-^ and the difference between the CSs across three criterion sessions and accompanying *t*-test results. Animal numbers correspond to the numbers in Figure 3A in the main text.

**
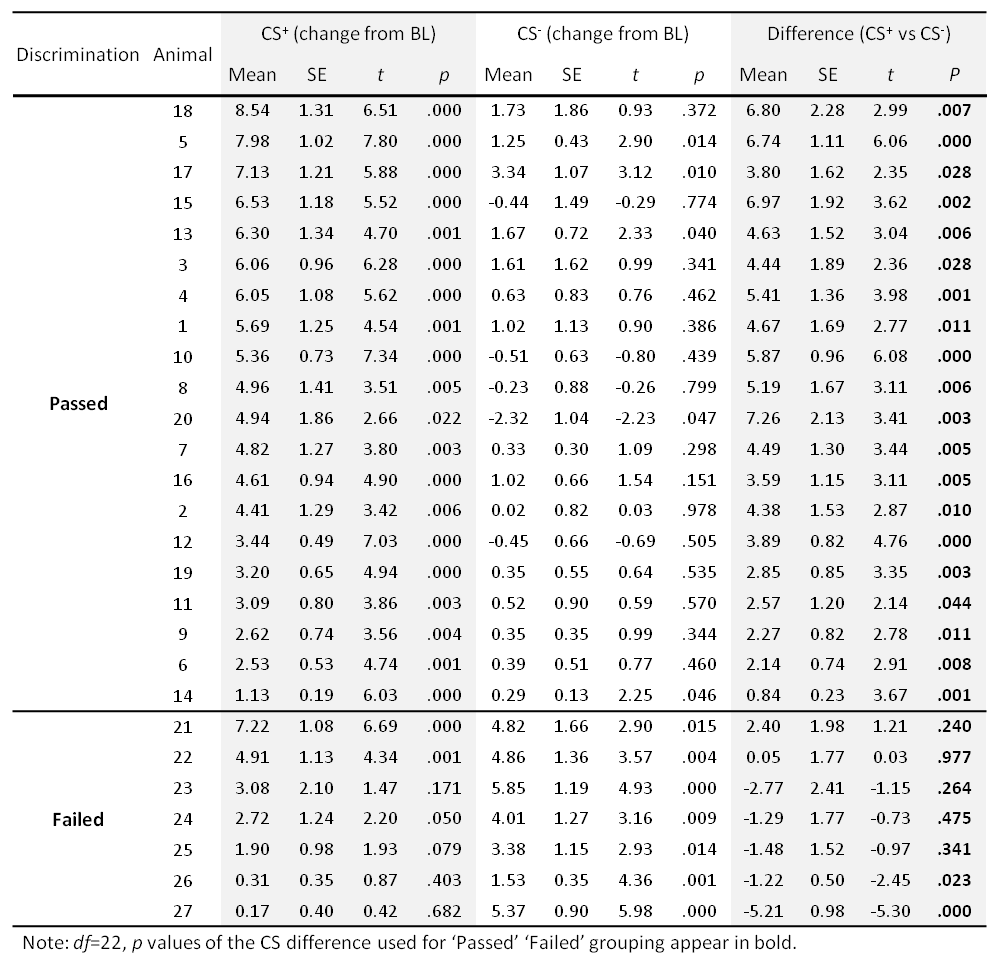
**

**Supplementary Table 2.** Individual animal’s mean HR for CS^+^, CS^-^ and the difference between the CSs across three criterion sessions and accompanying *t*-test results. Animal numbers correspond to the numbers in Figure 3A in the main text.


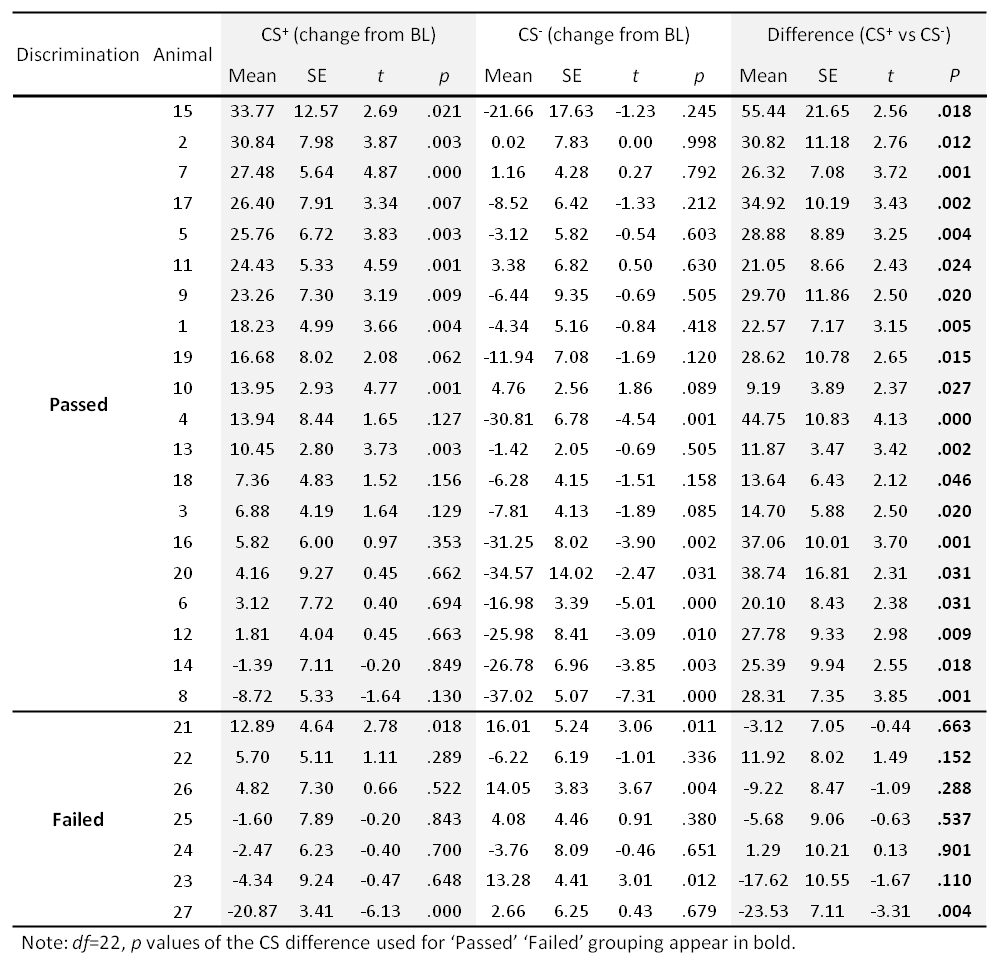


**Supplementary Table 3.** Comparison between the ‘passed’ and ‘failed’ groups for each of the seven measurements scored in the snake phase of the rubber snake test. *p<.05, **p<.01


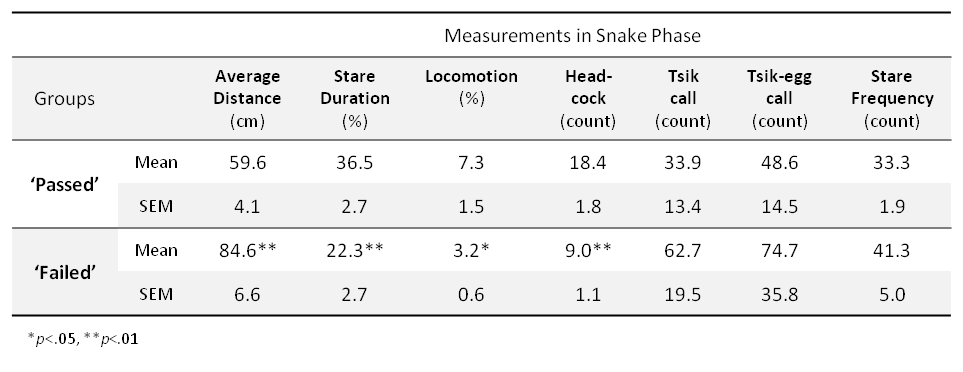


## Supplementary Figures


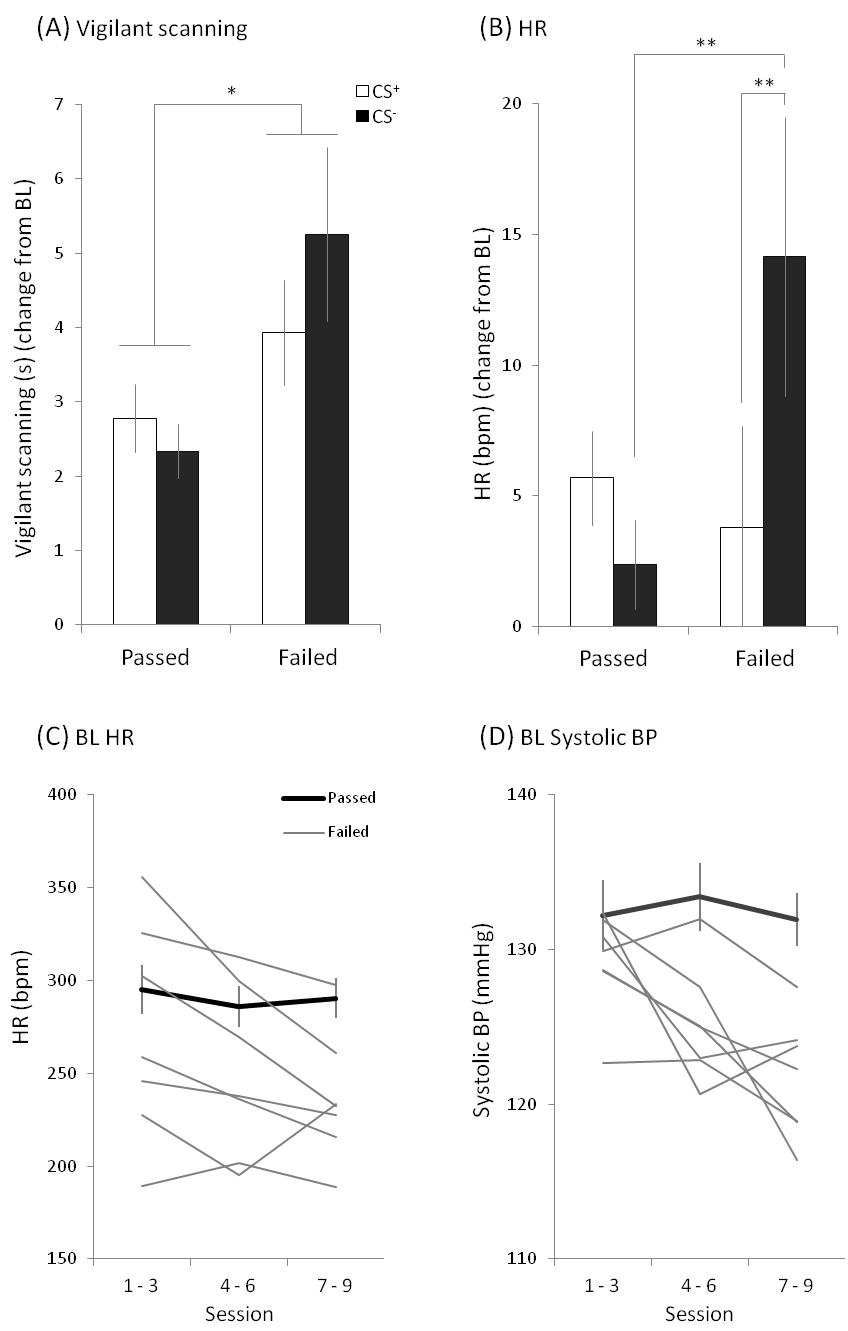


**Supplementary Figure 1.** (A, B) Mean (+/- SEM) behavioral and HR responses to the CS^+^ (open bar) and CS^-^ (filled bar), compared to BL, in the first three sessions of the animals in the ‘passed’ and ‘failed’ groups. (C, D) HR and BL Systolic BP across the first 9 sessions for the ‘passed’ group (mean, thick black line) and ‘failed' animals (individual animals, thin gray line). *p<.05, **p<.01


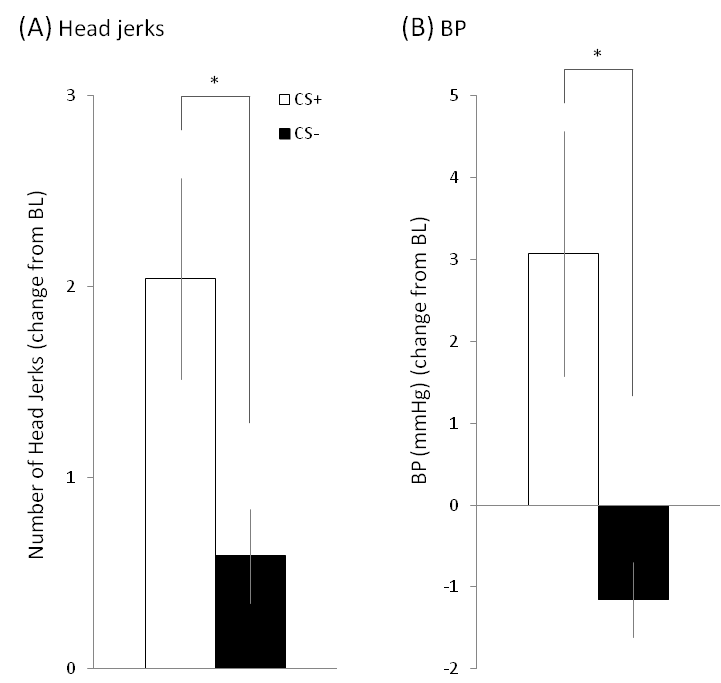


**Supplementary Figure 2.** Mean (+/- SEM) discriminative responses to the CS^+^ (open bar) and CS^-^ (filled bar), compared to BL, for (A) the behavior and (B) BP in the appetitive discrimination criterion sessions of the animals that were unable to discriminate the aversive CSs (‘failed’ group). **p*<.05

**
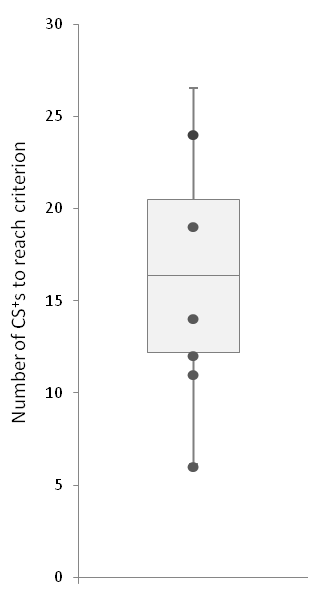
**

**Supplementary Figure 3.** The individual symbols in the graph indicate the number of CS^+^s to reach criterion on the appetitive task for each animal in the ‘failed’ group. Modified box plot depicts the number of CS^+^s to reach the criterion of the 23 animals from previous studies. The statistics include the mean (middle line), 95% confidence interval (upper & lower boxes) and 1 standard deviation (upper & lower whiskers). Notice that five of the six ‘failed’ animals were equivalent or better in learning the task than the 23 previously tested animals.


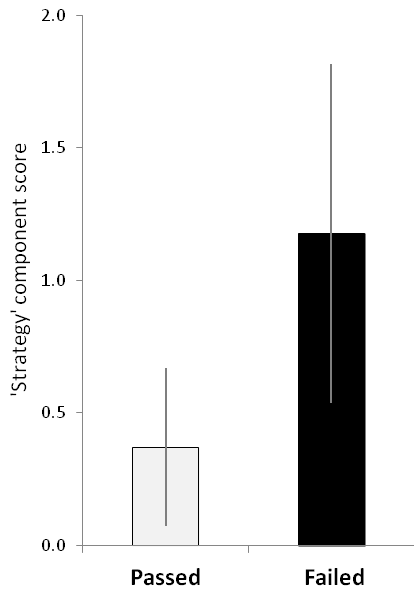


**Supplementary Figure 4.** Comparison of the mean (+/-SEM) of the ‘passed’ (n=7, open bar) and ‘failed’ (n=6, filled bar) groups for the ‘strategy’ component scores of the rubber snake test. The groups did not differ significantly from each other [*F*(1,11)=1.45, *p*=.25].


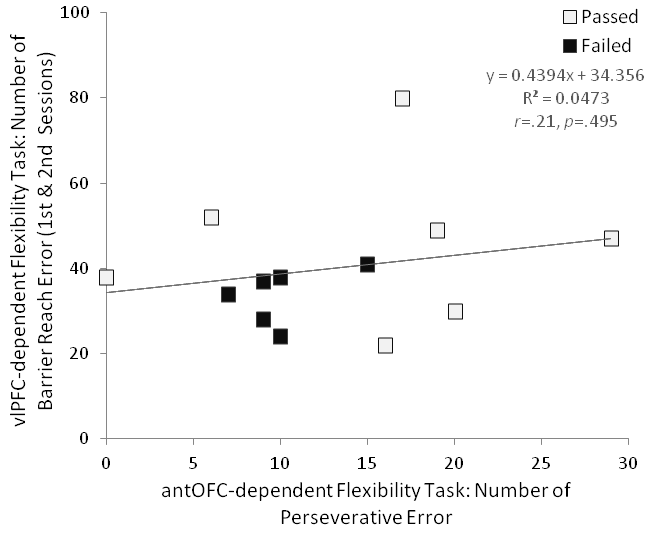


**Supplementary Figure 5.** The number of perseverative errors made on the vlPFC-dependent flexibility test and antOFC-dependent flexibility task. No significant correlation was found.


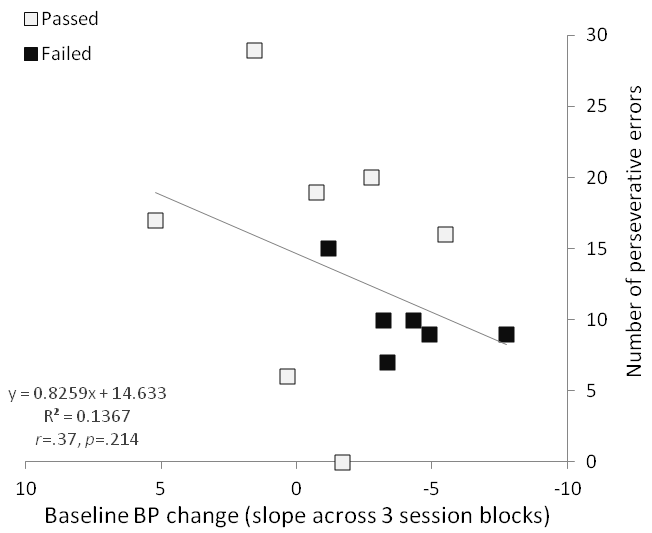


**Supplementary Figure 6.** Correlation between the number of perseverative errors in the antOFC-dependent flexibility test and the baseline BP change (slope across sessions 1-9) in the aversive discrimination task. The correlation is not significant.


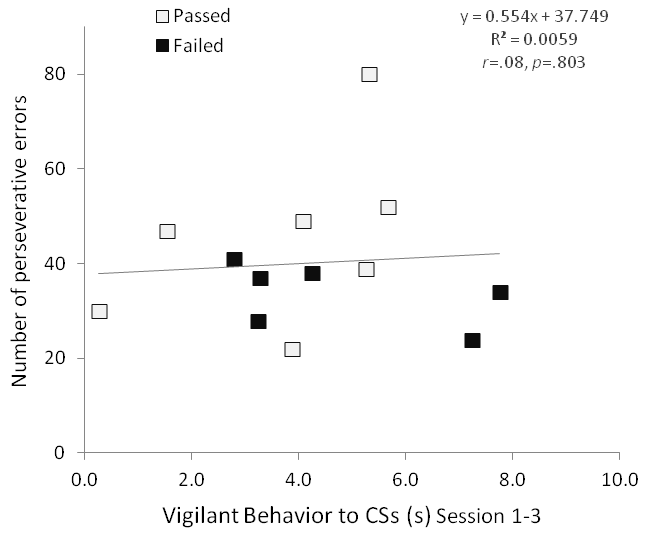


**Supplementary Figure 7.** Correlation between the number of perseverative errors (sum of barrier reaches in 1st and 2nd sessions) in the vlPFC-dependent flexibility test and the mean vigilant behavior scores to CSs during 1-3 sessions in the aversive discrimination task. The correlation is not significant.
